# Supplementary material for: Volcanism in slab tear faults is larger than in island-arcs and back-arcs
Source: Nat Commun. 2017 Nov 13;8:1451. doi: 10.1038/s41467-017-01626-w (PMC5682279; doi:10.1038/s41467-017-01626-w)
Supplement: Supplementary file 1 — Supplementary Information [file 41467_2017_1626_MOESM1_ESM.pdf]

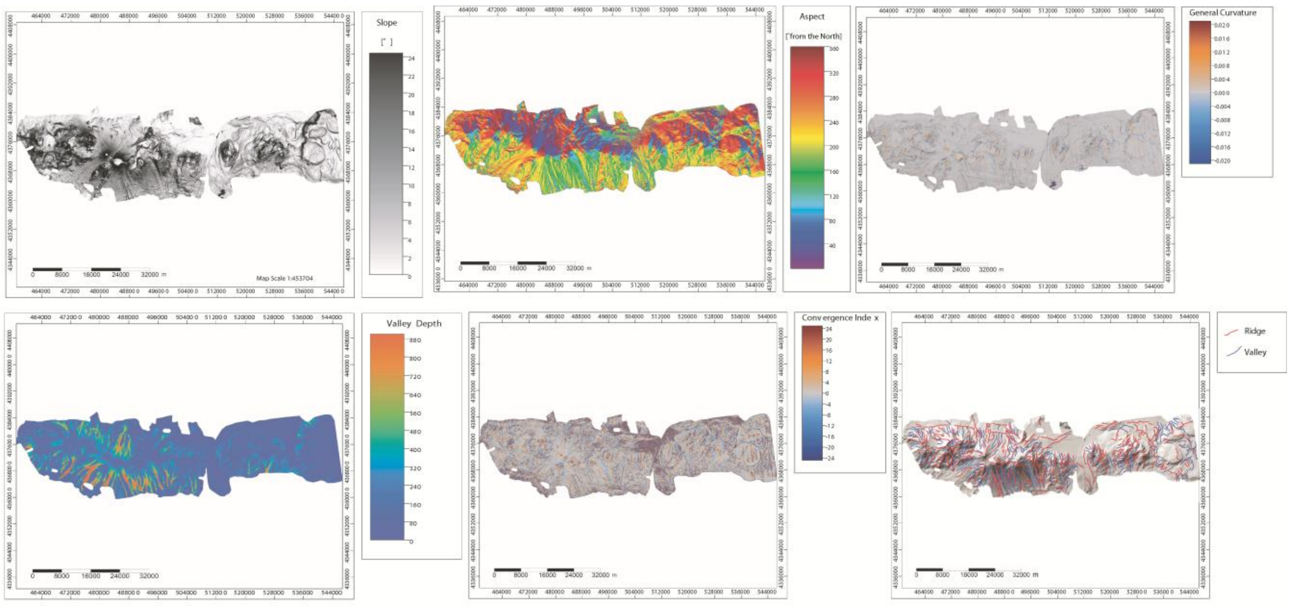

Supplementary Figure 1. Maps of morphometric parameters extracted from the seafloor DTM of STEPVC (see Supplementary Table 2 for the DTM specifications and Methods for details about the extracted parameters).

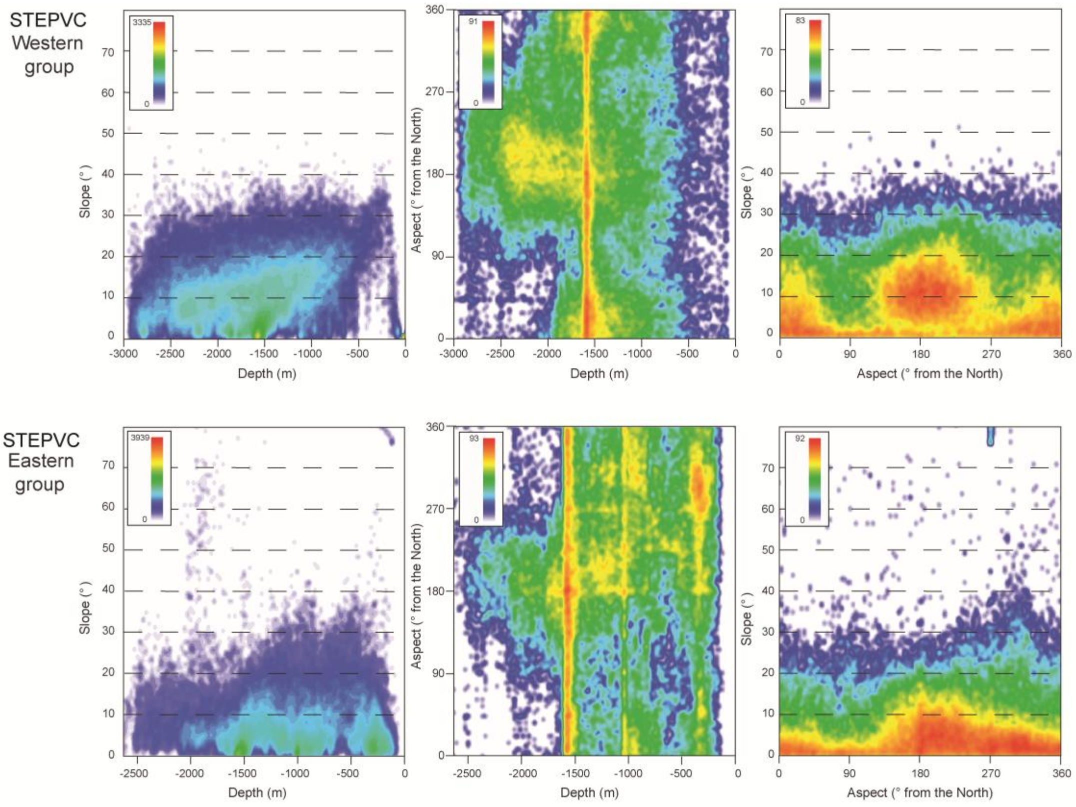

Supplementary Figure 2. Comparison among the DTM extracted parameters slope, aspect and depth of the seafloor in the STEPVC western and eastern groups.

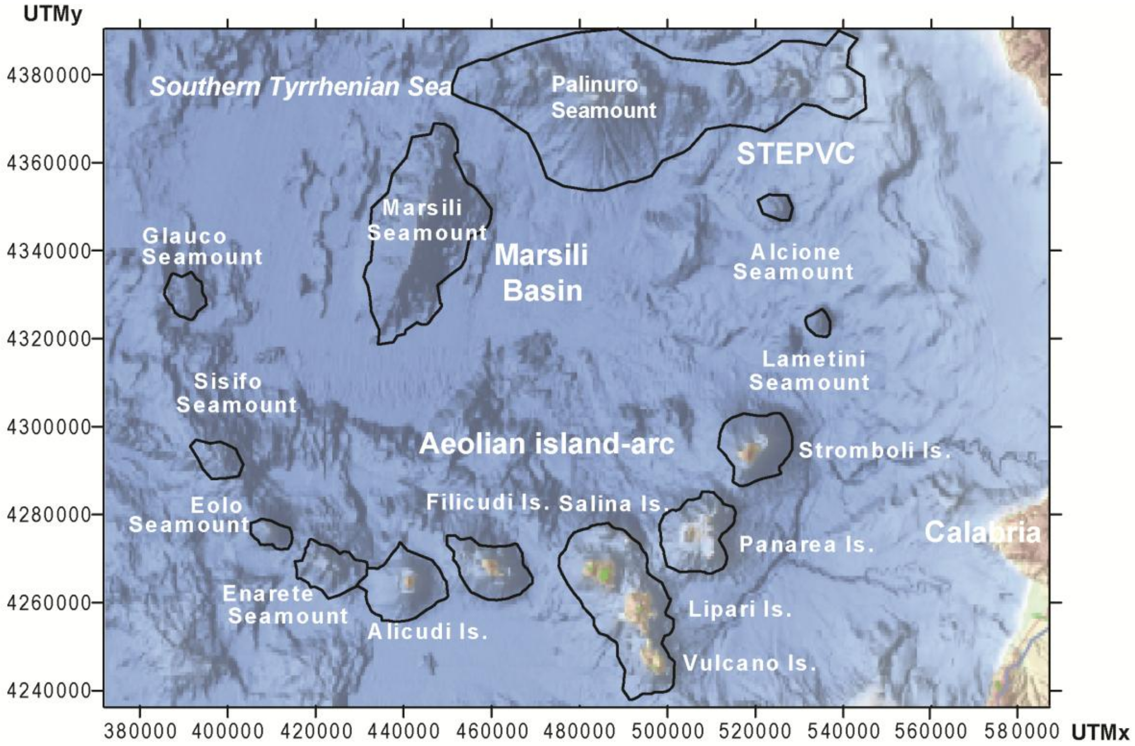

Supplementary Figure 3. Boundaries of the main volcanic edifices of the Southern Tyrrheanian Sea and Digital Terrain Model of the seafloor and islANDS (data from EMODnet Bathymetry Consortium 2016 EMODnet Digital Bathymetry at http://doi.org/10.12770/c7b53704-999d-4721-b1a3-04ec60c87238).

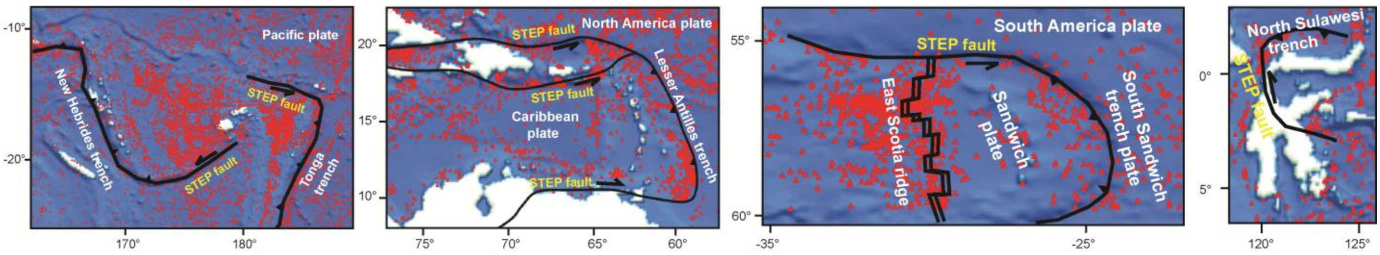

Supplementary Figure 4. Distribution of seamounts (red triangles) and STEP faults in the subduction zones of Tonga and New Hebrides, Lesser Antilles, Sandwich, and Sulawesi. The bathymetry is extracted from the global 30 arc-second GEBCO_2014 grid (http://www.gebco.net/data_and_products/gridded_bathymetry_data/gebco_30_second_grid/). The location of the seamounts is from Supplementary ref. 1; the geodynamic sketches and the kinematics of the STEP faults are from Supplementary ref. 2.

Supplementary Table 1. Echosounder and acquisition parameters

Oceanographic Cruise	Echosounder	Depth coverage	Swath opening	Fruequency (kHz)	Number of beams	DTM cell size
Aeolian_2007	Seabat 8150	-84/-2200	150°	50	126	20 m
Aeolian_2010	EM710		140°	40-100	400	5 m
SAFE_2015	Seabat 7150		140°	50	150	25 m

Supplementary Table 2. Statistical parameters of the Palinuro STEP seafloor digital terrain model (WGS 84, UTM zone 33N).

Grid Size:	1146 rows x 3586 columns
UTM x Minimum:	457025
UTM x Maximum:	546673
x spacing:	25
UTM y Minimum:	4358082
UTM y Maximum:	4386701
y spacing:	25
Maximum depth (m):	-3017
Minimum depth (m) :	-84
Mean:	-1387
Median:	-1430
Standard Deviation:	618
Range:	2932
Median Abs. Deviation:	415
Average Abs. Deviation:	499
Standard Error:	0.38
Skewness:	-0.13
Kurtosis:	2.51

Supplementary Table 3. Volume of the Southern Tyrrhenian Sea volcanoes. The volume of the emerged volcanoes includes their submerged portions.

Volcanic edifice(s)	Volume (km^3)
STEP Volcanic Chain	2704.82
Marsili Seamount ridge	856.26
Salina, Lipari, Vulcano, Panara and Stromboli emerged volcanoes	1859.23
Alicudi and Filicudi emerged volcanoes	231.85
Aeolian seamounts (Glauco, Sisifo, Enarete, Eolo, Lametini, Alcione)	463.71
Aeolian emerged volcanoes and seamounts	2554.79

Supplementary References

1. Hillier, J.K. & Watts A.B. Global distribution of seamounts from ship-track bathymetry data. *Geophys. Res. Lett.* **34**, L13304 (2007)

2. Govers, R. & Wortel, M.J.R. Lithosphere tearing at STEP faults: response to edges of subduction zones. *Earth Planet. Sci. Lett.* **236,** 505–523 (2005).
